# Food Insecurity and the Risk of Obesity, Depression, and Self-Rated Health in Women

**DOI:** 10.1089/whr.2020.0049

**Published:** 2020-08-31

**Authors:** Sydney K. Willis, Sara E. Simonsen, Rachael B. Hemmert, Jami Baayd, Kathleen B. Digre, Cathleen D. Zick

**Affiliations:** ^1^ Department of Epidemiology, Boston University School of Public Health, Boston, Massachusetts, USA.; ^2^ College of Nursing, University of Utah, Salt Lake City, Utah, USA.; ^3^ Department of Neurology and Ophthalmology and University of Utah, Salt Lake City, Utah, USA.; ^4^ Department of Family and Consumer Studies, University of Utah, Salt Lake City, Utah, USA.

**Keywords:** depression, food insecurity, obesity, self-rated health

## Abstract

***Background/Introduction/Objective:*** Recent studies have shown that food insecurity is associated with obesity, depression, and other adverse health outcomes although little research has been focused on these relationships in underrepresented cultural and social groups. In this study we elucidate the relationship between food insecurity, community factors, dietary patterns, race/ethnicity and health among underrepresented women.

***Materials and Methods:*** The data for this investigation come from a cross-sectional survey of women drawn from five urban Utah communities of color, including African immigrants/refugees, African Americans, Hispanics, American Indians/Alaska Natives, and Pacific Islanders, and women from four rural Utah counties. Multivariate logistic regression was used to assess the relationship between food insecurity and obesity risk, self-reported depression, and self-assessed health.

***Results:*** Urban women of color were more likely to report food insecurity than rural non-Hispanic white women. Obesity and depression scores were positively associated with food insecurity.

***Conclusions:*** Utah women of color had higher levels of food insecurity than reported in state or national data, highlight an important disparity. Nutritional education initiatives, evaluating food assistance programs, and screenings in clinical settings targeting specific racial/ethnic groups may help address the disparities observed in this study.

## Introduction

Food insecurity is defined as “limited or uncertain availability of nutritionally adequate and safe foods or limited or uncertain ability to acquire acceptable foods in socially acceptable ways.”^1^ Nationwide, food insecurity affected between 10% and 12% of households from 1995 to 2007 and increased to roughly 14% during the 2008–2009 recession. Levels have since returned to around 13%.^2^

Rates are higher for economically or socially disadvantaged populations. Households with minor children,^3^ older individuals,^3^ individuals living in rural areas,^2^ and women^4^ all experience higher rates of food insecurity, with women of color and rural women facing increased risks due to the intersection of these factors.^2,5^ Recent studies have shown that food insecurity is associated with obesity,^6^ depression,^7–9^ type 2 diabetes,^10,11^ and other adverse health outcomes.^12^

Although many studies have found an association between obesity and food insecurity, especially in women,^6^ the current literature lacks a detailed exploration of the relationship between food insecurity and health in underrepresented cultural and social groups, particularly among those with limited representation in prior research such as Pacific Islanders and African refugee and immigrant populations.^13,14^

The goal of this study was to elucidate the relationship between food insecurity, community factors, dietary patterns, race/ethnicity, and health among women in both rural and urban communities. We sought to examine (1) individual, community, and dietary factors associated with the presence of self-reported household food insecurity among women from five urban communities of color and rural non-Hispanic white women; (2) the association between obesity, depression, and self-perceived health and food insecurity in these groups.

## Materials and Methods

### Study population

This study was a cross-sectional analysis using data drawn from the Coalition for a Healthier Community for Utah Women and Girls (UWAG) study. UWAG was conducted through a partnership with Community Faces of Utah (CFU), an organization representing five urban communities of color, four rural communities, the University of Utah, the Utah State University Extension Program, and the Utah Department of Health. The larger study focused on assessing the effectiveness of a 12-month wellness coaching intervention on women's physical activity levels and dietary habits.^15^

Members of each respective community were trained as wellness coaches by the UWAG team to serve as community health workers focusing on preventive health. Wellness coaches recruited study participants at community events, including health fairs, churches, and cultural events, and through snowball recruitment techniques, where interested friends or family members of study participants were enrolled.

Once recruited, participants completed a baseline interview and wellness coaches collected anthropometric measures. After the collection of baseline measures, participants were randomized into a low- or high-intensity year-long intervention designed to promote healthy eating and physical activity. Detailed information about UWAG study methods and the cost-effectiveness of the intervention have been previously published.^15–17^ All study procedures were approved by the University of Utah Institutional Review Board and the Phoenix Area Indian Health Service Institutional Review Board. Verbal informed consent was obtained from all subjects/patients.

All data used in the current analysis were collected at baseline, before randomization. Rural, African American, Pacific Islander, and American Indian/Alaska Native (AI/AN) respondents completed an English survey. Hispanic respondents had the option of completing either an English or Spanish survey. African immigrant/refugee respondents from Burundi and Rwanda completed the survey in Kirundi.

Urban recruitment took place in Salt Lake County, a densely populated county in which one out of five residents speak English as a second language, and one out of eight residents are born outside of the United States.^18^ White non-Hispanic rural participants were recruited in San Juan, Beaver, Emery, and Duchesne counties. The population density of these counties ranges from 1.9 to 5.7 people per square mile in comparison to Salt Lake County, which has 33.6 people per square mile.^19^

### Measures

In-person, computer-assisted interviews were used to collect data on food insecurity, depression, and self-rated health. Food insecurity was assessed with the following question, which was adapted from a Behavioral Risk Factor Surveillance System (BRFSS) question that incorporated feedback from community partners: “How many days, in the past 30 days have you been concerned about having enough food for you or your family?” Response options included: “all of the days,” “most of the days,” “some of the days,” or “none of the days.” Food insecurity was determined to be present if a respondent answered “all of the days,” “most of the days,” or “some of the days” and absent if a respondent answered “none of the days.”^20^

All participants were screened for depression by using the Public Health Questionnaire-2 (PHQ-2) at baseline. Those scoring 3 or higher on the PHQ-2 were considered to have a positive depression screen.^21^ Data on self-perceived health came from a validated BRFSS question asked on the baseline survey: “How would you rate your health?” Response options included: “excellent,” “very good,” “good,” “fair,” or “poor.” Participants were categorized into either “poor” or “fair” self-rated health versus “excellent,” “very good,” or “good” self-rated health.

Anthropometric data included height, weight, waist circumference, hip circumference, and blood pressure. Wellness coaches were trained to take anthropometric measurements by using standardized approaches such as zeroing the scale before each measurement and removing the participants' shoes. Body mass index (BMI) was calculated based on body weight (kg) divided by height (cm) squared. Obesity was defined as BMI ≥32 for Pacific Islanders and BMI ≥30 for all other groups.^22–24^

In-person, computer-assisted interviews were also used to collect data on demographics, health status, health care access, health literacy, diet and exercise behaviors, attitudes, quality of life, social support, and environmental factors.

Community membership included self-identified membership as AI/AN, Hispanic, Pacific Islander, African immigrant or refugee, African American, or rural non-Hispanic white. Income was categorized based on the percent of the federal poverty level (FPL) (<100, 101–130, 131–185, >185% FPL, and not reported). Age (18–29, 30–39, 40–49, and ≥50 years) and education (<high school, high school graduate, some college, and ≥college graduate) were self-reported by participants during the baseline interview.

Information on food consumption was collected by asking weekly intake of fast food, soda, orange vegetables, green vegetables, beans, “other” vegetables (including tomato, corn, eggplant, peas, lettuce, cabbage, and white potatoes), water, juice, and fruit intake by using questions from the BRFSS survey and classified into consumption of ≥5 fruit and vegetables per day (yes vs. no).^21^

Information on physical activity was ascertained by asking, “In an average week, how much time do you spend being physically active or doing exercise?.”^25^ The threshold for receipt of adequate physical activity was based on the CDC's definition of meeting guidelines: 2.5 hours or more per week of moderate to vigorous physical activity.^25^

Residential addresses of study participants were geocoded by using a Geographic Information System (GIS). Geocoded addresses were overlaid onto census tract level data provided by the Food Access Research Atlas (FARA).^26^ FARA, developed by the Economic Research Service of the United States Department of Agriculture, is a collection of spatial food insecurity variables including the location of low-income census tracts, food deserts, and census tracts that have limited access to food distributors such as grocery stores.

FARA defines low-income census tracts as any tract with one or more of the following characteristics: “poverty rate is 20 percent or greater; the tract's median family income is less than or equal to 80 percent of the State-wide median family income; or the tract is in a metropolitan area and has a median family income less than or equal to 80 percent of the metropolitan area's median family income.”^27^ Limited access census tracts are defined by distance to the nearest grocery store or supermarket. Distances greater than or equal to ½ mile in urban areas and 10 miles for rural tracts are considered low access. Food deserts are census tracts that are both low income and low access.

We used analysis of variance and chi-square tests to assess differences in dietary factors between individuals with self-reported food insecurity within the past 30 days (some and most or all days) and individuals without food insecurity in the past 30 days. We used multivariable logistic regression to obtain the odds of having any food insecurity compared with having no food insecurity and 95% confidence intervals (95% CI). For the multivariable logistic regression, we combined levels of any reported food insecurity in the past 30 days (compared with no reported food insecurity) due to small counts for those who reported food insecurity for most or all of the past 30 days.

Two multivariate models were estimated: Model A with mutual adjustment for other health outcomes (BMI, positive depression score, self-rated health); Model B with adjustment for covariates in model A as well as demographic and community factors including community group, minor children in the home, percent of FPL, residing in a food desert, age, education, meeting physical activity guidelines, and consumption of five or more fruits/vegetables per day. As we compared three health outcomes, we applied the Bonferroni correction to p-values for the main health outcomes, using a *p*-value of 0.017 as our alpha level. All covariates and interactions included in the multivariate regression were determined *a priori* and were assessed for collinearity. No covariates included in the models were determined to be collinear.

Data were analyzed by using StataSE14 (College Station, TX), SAS version 9.4 (SAS Institute, Cary, NC), qGIS 2.18.9, and ArcGIS 10.x (Redlands, CA).

## Results

A total of 516 women enrolled in the study and completed the baseline interview. The racial/ethnic make-up of participants was 15% African Immigrant/Refugee, 19% African American, 26% Hispanic, 16% Pacific Islander, 14% AI/AN, and 10% rural non-Hispanic white. The average age of the participants was 42 years old, and the majority of participants had children younger than the age of 18 living at home. More than two-thirds of the participants had a household income less than 185% FPL adjusted for household size (Table 1) with African, Hispanic, and AI/ANs having the largest proportion of respondents with incomes <185% of the FPL (data not shown). The modal educational level was “some college.” Slightly more than one-fifth of participants lived in a food desert as defined as census tracts that are both low income and low access.

**Table 1.** Demographic Characteristics of 516 Utah Women and Girl Study Participants at Baseline

	Total
Age (years), mean	41.5
Group, %	
Urban African immigrant/refugee	14.7
Urban African American	19.6
Urban Hispanic	25.6
Urban Native Hawaiian/Pacific Islander	16.5
Urban American Indian/Alaskan Native	14.2
Rural non-Hispanic white	9.5
Income as a percent of the FPL, %	
≤100%	41.5
101%–130%	12.8
131%–185%	15.3
>185%	23.1
Not reported	7.4
Minor children in home, %	65.9
Education level, %	
<High school graduate	10.7
High school graduate	25.6
Some college	39.2
≥College graduate	24.6
Living in food desert^a^, %	21.3
Self-rated health, %	
Excellent	5.0
Very good	14.0
Good	37.0
Fair	33.3
Poor	10.7
Positive PHQ-2 depression screen^b^, %	21.1
BMI, %	
Underweight/normal weight	16.9
Overweight	20.4
Obese	62.8
Food insecurity in past 30 days, %	
None	68.8
Some days	24.4
Most days	3.7
All days	3.1

^a^ Food Desert defined as low income and low access tract measured at 1 mile for urban areas and 10 miles for rural areas.

^b^ Positive screening defined as PHQ-2 score ≥3

BMI, Body mass index; FPL, federal poverty level; PHQ-2, Public Health Questionnaire-2.

The health indicators in Table 1 suggest that study participants experience a number of health challenges, as 44% rated their overall health as either fair or poor and 20% had a positive PHQ-2 depression screen. Slightly more than 83% were overweight or obese.

Among all participants, 69% reported no food insecurity within the past 30 days; 24% reported food insecurity on some days; 4% reported food insecurity on most days; and 3% reported food insecurity during all of the past 30 days. The proportion of respondents reporting “some” and “most or all” days of food insecurity across communities was as follows: AI/AN (32% and 12%, respectively), Hispanic (32% and 10%, respectively), African Immigrant/Refugee (30% and 1%, respectively), Pacific Islander (25% and 8%, respectively), African American (14% and 5%, respectively), and rural Non-Hispanic white (6% and 0%, respectively). Reported levels of food insecurity (19%–43%) among the communities of color were higher than both the state (12%) and the national average (13%).^2^

When examining dietary and physical activity behaviors associated with food insecurity (Table 2), average consumption of fruit and vegetables per day and participation in at least 2.5 hours per week of moderate-to-vigorous physical activity did not differ between those with and without food insecurity.

**Table 2.** Association Between Self-Reported Food Insecurity in the Past 30 Days and Dietary and Physical Activity Characteristics

	No food insecurity reported in past 30 days (n = 355)	Food insecurity reported in some of the past 30 days (n = 126)	Food insecurity reported in most or all of the past 30 days (n = 35)	p
Consumption of 5+ fruits and vegetables per day, *n* (%)	100 (28.2)	38 (30.2)	9 (25.7)	0.85
Meets physical activity guidelines^a^, *n* (%)	127 (35.8)	34 (27.0)	12 (34.3)	0.20
Weekly mean (SD) other vegetable intake	5.0 (6.4)	3.8 (5.5)	2.9 (3.1)	<0.05
Weekly mean (SD) orange vegetable intake	2.7 (5.1)	2.0 (3.4)	3.5 (6.9)	0.19
Weekly mean (SD) green vegetable intake	5.0 (8.1)	4.3 (5.5)	2.3 (2.6)	<0.05
Weekly mean (SD) bean intake	2.0 (3.9)	2.6 (4.2)	1.3 (1.5)	<0.05
Weekly mean (SD) water intake	36.7 (23.2)	36.1 (21.5)	39.5 (28.4)	0.80
Weekly mean (SD) juice intake	3.1 (6.4)	3.3 (8.7)	4.7 (10.4)	0.67
Weekly mean (SD) fruit intake	11.9 (9.4)	12.4 (11.7)	9.9 (13.5)	0.44
Weekly mean (SD) fast food intake	2.0 (4.5)	1.7 (4.5)	2.3 (5.9)	0.67
Weekly mean (SD) soda intake	4.5 (8.5)	4.9 (6.8)	5.9 (9.6)	0.64

^a^ Meets CDC guidelines defined as ≥2.5 hours per week of moderate to vigorous physical activity.

SD, standard deviation.

When stratified by individual components of diet, weekly consumption of orange vegetables, fruit, 100% fruit juice, or water did not substantially differ by food security status. Weekly consumption of “other” vegetables (including tomato, corn, eggplant, peas, lettuce, cabbage, and white potatoes), green vegetables, and beans significantly differed between food security groups. Food secure individuals consumed an average of 5.0 (SD: 6.4) “other” vegetables per week, individuals with some food insecurity in the past 30 days consumed 3.8 (SD: 5.5) “other” vegetables per week, and individuals with food insecurity in most or all of the past 30 days consumed “other” vegetables 2.9 (SD: 3.1) times per week (*p* < 0.05). Those without food insecurity consumed dark green vegetables at higher levels that those with food insecurity (5.0 vs. 4.3 [some] vs. 2.3 [most or all], respectively, *p* < 0.05).

In unadjusted analyses (Table 3), food insecurity risk was associated with all health measures (including obesity, a positive depression score, and poor or fair self-rated health), being an urban woman of color compared with a non-Hispanic rural white, the presence of children in the home, low income, and residence in a food desert.

**Table 3.** Multivariable Logistic Regression Results for Self-Reported Food Insecurity During Some, Most, or All of the Past 30 Days Versus No Food Insecurity in the Past 30 Days (*N* = 516)

Characteristic	Unadjusted		Adjusted Model A		Adjusted Model B	
	OR	95% CI	OR	95% CI	OR	95% CI
BMI						
Normal weight/overweight	1.00	Reference	1.00	Reference	1.00	Reference
Obese	1.54	1.04–2.29	1.38	0.91–2.09	1.71	1.08–2.71
PHQ-2 depression score						
Negative	1.00	Reference	1.00	Reference	1.00	Reference
Positive	2.26	1.46–3.49	1.93	1.23–3.04^*^	2.12	1.29–3.50^*^
Self-rated health						
Excellent, very good, good	1.00	Reference	1.00	Reference	1.00	Reference
Fair, poor	2.09	1.43–3.06	1.72	1.15–1.26^*^	1.35	0.87–2.10
Community						
Rural non-Hispanic white	1.00	Reference	NA		1.00	Reference
Urban American Indian/Alaskan Native	11.97	3.41–42.02			8.84	2.37–33.02
Urban Hispanic	10.95	3.24–37.02			7.38	2.02–26.89
Urban Native Hawaiian/Pacific Islander	7.53	2.15–26.35			8.38	2.20–31.94
Urban African Immigrant/Refugee	7.07	2.00–25.05			4.35	1.11–16.70
Urban African American	3.55	1.00–12.65			4.70	1.26–17.58
Children in home						
No	1.00	Reference	NA		1.00	Reference
Yes	1.72	1.14–2.59			1.04	0.61–1.77
FPL, % of FPL						
<100%	5.55	2.98–10.31			5.15	2.46–10.68
101%–130%	4.01	1.89–8.52			3.54	1.55–8.07
131%–185%	3.08	1.47–6.45			2.71	1.23–5.96
>185%	1.00	Reference	NA		1.00	Reference
Not reported	2.68	1.08–6.67			3.31	1.22–8.97
Food desert						
No	1.00	Reference	NA		1.00	Reference
Yes	1.65	1.06–2.55			1.51	0.93–2.47
Age, years						
18–29	1.00	Reference	NA		1.00	Reference
30–39	1.36	0.81–2.31			1.24	0.69–2.25
40–49	1.40	0.81–2.41			1.35	0.71–2.56
≥50	0.77	0.44–1.33			0.97	0.50–1.90
Education						
Less than high school	1.00	Reference	NA		1.00	Reference
High school graduate	1.08	0.56–2.09			1.69	0.81–3.55
Some college	0.90	0.48–1.69			1.70	0.82–3.51
≥College	0.56	0.28–1.12			1.50	0.67–3.33
5+ Fruit and vegetable servings per day						
No	1.00	Reference	NA		1.00	Reference
Yes	1.05	0.70–1.59			1.17	0.73–1.86
2.5+ Hours of weekly exercise						
No	1.00	Reference	NA		1.00	Reference
Yes	0.72	0.48–1.08			0.88	0.55–1.40

^*^ Statistically significant with Bonferroni correction applied.

In the fully adjusted multivariate model, individuals who were obese had an increased odds of reporting food insecurity compared with individuals of normal weight or overweight (OR: 1.71, 95% CI: 1.08–2.71), although results were not statistically significant when applying the Bonferroni correction. Individuals with a positive PHQ-2 depression score had 2.12 (95% CI: 1.29–3.50) times the odds of reporting food insecurity compared with those with a negative PHQ-2 depression score.

Other factors associated with food insecurity in the fully adjusted model included being an urban woman of color, with all groups having an increased risk of food insecurity compared with rural non-Hispanic whites as well as FPL. A dose-response relation was observed among individuals with lower income having a higher odds of food insecurity compared with individuals with higher income levels. Although no statistically significant association was observed, those with fair or poor self-rated health had slightly increased odds of reporting food insecurity compared with those with excellent, very good, or good self-rated health (OR: 1.35, 95% CI: 0.87–2.10).

## Discussion

In this cross-sectional study investigating food insecurity among a group of diverse women of color and rural non-Hispanic whites, we found associations between both obesity and food insecurity as well as between depression and increased odds of experiencing food insecurity. These associations are congruent with findings from other studies.^6–9^ Food insecurity among women is important, as female study respondents are generally more likely to report household food insecurity when compared with male respondents.^28^ A 2017 meta-analysis found that female-headed households were 75% more likely to be food insecure than male-headed households (95% CI 49–96).^28^

Although the relationship between poverty and food insecurity among women has been well established,^29–32^ food insecurity specifically among women of color, holding income constant, is emerging in the literature. Women from Pacific Islander, AI/AN, and refugee communities are currently underrepresented in the literature. Our findings add to this literature by highlighting a broader range of racial/ethnic communities.

Many of the communities in this study have not been traditionally included in research, highlighting that these groups may have unique risk profiles. These differences suggest that there might be a need for culturally tailored nutrition education programming focused on reducing food insecurity for these high-risk groups.^33^

It may be instructive to identify how race/ethnicity and food insecurity interact to increase the risk of poor health outcomes in such groups. Unfortunately, sample sizes for these groups do not allow for exploration of such interactions in the current study, indicating a need for future research with these populations.

In addition, our small sample size precludes us from making conclusions about differences in food insecurity between any groups other than indicating a statistically significant difference in food insecurity among rural whites and other racial/ethnic groups included in our study. Future work should include qualitative methods such as personal interviews and focus groups with members of different communities to gain a better understanding of barriers and facilitators to decreasing food insecurity, particularly in under-studied communities.

The association between food insecurity and obesity has been demonstrated in previous studies.^6,34–39^ Three studies that focused on communities of color found an association between food insecurity, poor diet, and obesity ^35,36,40^ whereas only one found no association.^39^

It has been hypothesized that the mechanism behind this finding is that either the consumption of low-cost, calorie-dense, nutrient-deprived food by those with food insecurity leads to obesity or fattening is a physiological response to a threatened food supply.^6^

Although we saw an association between obesity and food insecurity and found no appreciable association between food insecurity and self-reported intake of five or more servings of fruit and vegetables per day, frequency of fast food consumption, frequency of soda consumption, or intake of some specific types of fruits and vegetables, we did observe a difference between intake of green vegetables, beans, and other vegetables.

It should also be noted that although our data indicate that individuals with and without food insecurity are eating similar quantities of most fruits and vegetables, we were unable to evaluate the quality and source of these foods, such as fresh versus canned or frozen fruits and vegetables.

Our finding of a positive association between food insecurity and depression has been corroborated by other studies.^7–9,41,42^ One prior study observed that depression and anxiety are more common among food insecure individuals, particularly among women.^8^ A study that examined depression among Supplemental Nutrition Assistance Program (SNAP) participants found that very low food security was associated with higher odds of depression in both participants and eligible nonparticipants.^42^ Although we are unable to determine the direction of this association in this analysis, it is likely that the relationship is bidirectional: Food security may affect mental health as a stressor ^41^ and depression may have negative impacts on a woman's ability to work, and/or obtain and prepare food, thus contributing to food insecurity.^43,44^

As of 2016, 12% of Utah residents reported being food insecure, which is near the national average of 13%.^2,45^ Our findings indicate that even in a state with average levels of food insecurity, striking disparities may be present in subgroups as the rate of food insecurity in our sample was 30%.

A strength of our work is the inclusion of urban women of color from multiple communities and the inclusion of non-Hispanic white rural women. We were also able to differentiate between African immigrants and refugees compared with African Americans, distinct groups that are traditionally analyzed as a single group.^44^ In addition, the utilization of wellness coaches for participant recruitment enabled us to reach diverse and historically underserved study participants. That said, we also acknowledge that the sample in the current study is not likely fully representative of these groups due to convenience sampling methods and the geographic restrictions of the study.

Limitations of our study include lack of an urban, non-Hispanic white comparison group and relying on self-reported information for dietary composition. The lack of an urban non-Hispanic white comparison group limits the ability to compare the odds of food insecurity for these community groups with the dominant racial/ethnic group in the area. The reference group utilized was rural non-Hispanic whites, who have a higher incidence of food insecurity than urban non-Hispanic whites; thus, the odds observed in this study are likely an underestimate of the odds compared with urban non-Hispanic whites.

Self-reported data might be subject to social desirability reporting or error in recall, particularly for foods that are eaten rarely. There is a possibility that the study results may be affected by dependent misclassification as food insecurity and health outcomes, specifically self-reported health and depression, were assessed at the same time point and using the same instrument, a self-report questionnaire.^46^

In addition, this study assessed food insecurity status based on a single question that restricted the report of food insecurity to the past 30 days. Recent work is utilizing more detailed instruments that may provide a more accurate ascertainment of food insecurity. The question utilized in this study, however, was adapted from a BRFSS question that had been previously used in Utah and allows for comparison to local and national data.^47^ In addition, to achieve sufficient statistical power in the multivariable analyses we combined all levels of food insecurity (all, most, and some days) for the past 30 days, which limited our ability to observe the association between health and more detailed levels of food insecurity.

Our findings are potentially impacted by residual confounding, as we did not have information about types of food supports that some study participants may have had access to, including SNAP benefits, food banks, or other religious or community food assistance programs.

Lastly, because of the cross-sectional nature of our data, we are unable to determine causality or temporality. The relationship between food insecurity and health are likely bi-directional and complex. Although our study design allowed us to evaluate the differences in food security and the disproportionality of the studied health conditions among women of color, future longitudinal studies are recommended.

The role of food assistance programs in mitigating food insecurity among our sample was not investigated, as gathering data on program participation was not part of the original study design. However, we know that among all SNAP participants, slightly less than one-quarter remain food insecure.^48^ Moreover, research has found that reports of food insecurity among SNAP recipients increase, and that healthy eating awareness declines, with the number of days that have elapsed since a household's receipt of its monthly SNAP benefit.^49,50^

Qualitative research also reveals that SNAP recipients' coping strategies toward the end of the month often include skipping meals and seeking instrumental food supports from family and friends.^51^ These findings coupled with the relatively high rates of food insecurity observed in our targeted community samples and differences in the intake of some food types suggest that an investigation of the potential interplay between community group membership and the structure of food assistance programs could be an important policy-relevant area for future research.

Screening for social determinants of health is becoming more common in clinical settings, and, as such, screening for food insecurity is increasing. With the implementation of screening, clinicians will undoubtedly encounter patients with food insecurity and other key social determinants such as poverty, violence, unemployment, and unstable housing.^52^ Our findings suggest that these routine screening for social determinants of health should include food insecurity questions. The health care system can play an important role in helping link patients with social services to address their unique social determinant needs.

## Conclusions

In this study of five distinct groups of women of color and rural non-Hispanic whites in Utah, we found that self-reported food insecurity was associated with depression, obesity, income, and racial/cultural group membership in multivariable models. This study was one of the first to target under-represented racial/ethnic groups such as African refugees/immigrants and Pacific Islanders.

Our findings indicate a need to investigate why certain health factors and racial/cultural group membership are linked to relatively high risks of food insecurity. Our findings of persistent differences in food insecurity by ethnic group suggest that culturally targeted efforts, elucidating availability and utilization of food assistance programs, and screenings in clinical settings may be needed to address food insecurity in these high-risk groups.

## Abbreviations Used

AI/AN: American Indian/Alaska Native
BRFSS: Behavioral Risk Factor Surveillance System
CI: confidence interval
FARA: Food Access Research Atlas
FPL: federal poverty level
GIS: Geographic Information System
PHQ-2: Public Health Questionnaire-2
SNAP: Supplemental Nutrition Assistance Program
UWAG: Utah Women and Girls
